# Progress in Medicine

**Published:** 1895-04-27

**Authors:** 


### Progress in Medicine.

### DIETETICS.

**Milk.**—M.M. Gilbert and Dominici[1] have furnished fresh evidence of the antiseptic power of milk. An adult man, whose fæces contained 67,000 germs per milligramme, was restricted for five days to milk. At the end of two, three, four, and five days the germs had fallen to 14,000, 5,000, 4,000, 2,500 respectively. These workers also showed that a dog's stomach contained at an advanced period of digestion 50,000 germs per milligramme, and the duodenum about 30,000, while in the ileum the number, towards the ileo-cæcal valve, increased to 100,000. In a dog fed for two weeks on milk, and killed two hours after a meal of 300 grammes of milk, the stomach contained 100 germs per milligramme, the duodenum 50, and the ileum 1,300. While milk, however, is thus of great value in typhoid fever, in some forms of diarrhœa, and in Bright's disease, there seems to be little doubt that children become infected with summer diarrhœa through their milk food. This being the case, sterilisation is advisable. Dr. Rowland Freeman[2], of New York, describes an interesting and novel kind of dispensary, the expenses of which have been defrayed by one charitable gentleman. In this dispensary sterilised milk is supplied to mothers on the payment of a small sum. Before the cow's milk is sterilised it is diluted with water, and sugar of milk added, in order to approximate it in character to human milk. It is then poured into bottles which have been sterilised at a heat of 302 deg. Fahr., and afterwards cooled. The filled bottles are kept in water at a temperature of 167 deg. Fahr. for half-an-hour, then cooled and stored ready for distribution. Thirty-four thousand eight-ounce bottles were supplied in one season. The *Therapeutic Gazette*[3] gives a brief article upon the relative value of boiled and raw milk as food. Some experiments of Dr. Randolph, of Philadelphia, are quoted, in which an equal number of healthy men were given boiled and raw milk. After a certain time all were made to vomit by the administration of hypodermic injections of apomorphine. In every instance it was found that the raw milk was more digested than the cooked. Crolas, on the other hand, is quoted as believing that boiled milk is superior to raw. The writer of the article suggests that in the healthy raw milk is more easily digested, but that in some forms of diarrhœa the boiled is more desirable. Various methods of making milk more palatable or digestible are referred to by different writers. Dr. Edes,[4] of Boston, Mass., describes the following. A pint of milk is gently warmed, and into it is dropped very slowly and with constant stirring about twenty minims of the dilute hydrochloric acid. In this way a whey is produced with a fine flocculent coagulum floating in it. The casein instead of being separated out is retained. Milk prepared in this way has not the unpleasant bitter taste of pancreatised milk, yet has lost the flat taste of ordinary milk objectionable to many. Bovet[5] of Pougues states that by adding a vegetable ferment, called légumine, milk is not only made digestible, but may be taken when all other foods will be rejected. Légumine is a vegetable ferment which acts upon the casein, turning it into a soluble albuminoid. Borysowski[6] writes upon the value of aerated milk. He states that in St. Petersburg liquid $CO_2$ stored in iron bottles can be obtained from special factories. Patients can aerate their own milk at home. They are supplied with an apparatus that consists of two tubes soldered together, passed through a tight-fitting cork. One tube is long, and passing down into the milk conveys the $CO_2$ when connected with the iron bottle; the other tube is short for the escape of air.

**Bread.**—New bread has generally been condemned as unwholesome, but Dr. Troitzki[7] states that new bread contains no micro-organisms, but that when cut and allowed to be uncovered, harmless and pathogenic microbes find it an excellent nutrient medium. Drs. Waldo and Walsh,[8] however, have shown that at least non-pathogenic micro-organisms can survive the heat of the baker's oven, and they argue that if these harmless microbes can withstand that heat, pathogenic may also, and that c[illegible]mination of the dough with water containing typhoid bacilli might lead to infection with the disease. Dr. Bardet,[9] of Paris, speaks of the tendency to the return to the use of wholemeal bread. He considers that this tendency should be encouraged on dietetic and therapeutic grounds. Bread made in this way contains 40 instead of 20 per cent. of gluten, twice as large a proportion of certain salts, particularly the assimilable phosphates are present, and it also retains laxative fatty matter, which is removed in white bread. While it would be a mistake to recommend it to those who are accustomed to an abundant meat diet, it is of value to vegetarians and to those of the poorer classes who need some addition to their nitrogenous food. It is also of value to those who are constipated by temperament, on account of the presence of laxative wheat oil.

**Sugar.**—Vaughan Harley[10] has experimented upon himself as to the value of sugar as food. He found that the amount of work done on a sugar diet alone for one day was nearly equal to that done on a full diet. Other experiments were performed in the way of combining large quantities of sugar with small diets of other articles, and all tended to show sugar to be a powerful producer of muscle energy. Dr. Harley thinks sugar might be used profitably as an important item of food in those who have good digestions. Dr. Eccles (of Brooklyn)[11] speaks of the digestion of sugar, and compares the value of maltose and glucose. Maltose and cane sugar cannot be assimilated as such, but have to be transformed into dextrose. Starch in the mouth is mixed with saliva, the ptyalin of which commences there to convert it into maltose, a process that is continued for some time in the stomach; this maltose is not absorbed as such, but in the small intestine is transformed into dextrose. Glucose can be absorbed from the stomach, and Dr. Eccles considers it harmful. In those people who do not masticate their food well the starch is not mixed sufficiently with saliva, maltose is not produced, and dyspepsia results. Dr. Eccles says a tea-spoonful of malt extract containing diastase is of great value in such cases. The contents of the stomach do not become sufficiently acid to stop the sugar-forming process until some little time after food has been taken.

**Poisonous Foods.**—During the months of June, July, and August, 1894, several cases of poisoning by various forms of food occurred in different parts of England. From sixty to seventy persons suffered, and there were five deaths. The deaths occurred after eating meat, pork, fried fish, brawn, and tinned salmon. In only one instance, in the case of the fried fish, was there any complaint of peculiar taste in the article of diet. Dr. Luff[13] has read a paper before the Chemists' Assistants' Association upon poisonous and infected foods. In that paper he describes the symptoms of illness produced by such foods, and then proceeds to speak upon the nature of the poisons. The symptoms may be mainly gastric and intestinal, such as abdominal pain, vomiting, and diarrhœa, or nervous symptoms may be more pronounced, such as muscular weakness, drowsiness, and even insensibility. Dr. Luff refers to an epidemic that occurred in Middlesbrough in 1888, due, apparently, to the consumption of American bacon prepared in an unsatisfactory way. There were no fewer than four hundred and ninety deaths. The food may be poisonous owing to the presence of a micro-organism, or of some organic chemical poison which may be a ptomane, albumose, or toxin. The symptoms induced by poisonous foods are not always due to chemical poisons previously produced in the article consumed, since virulent micro-organisms have been found in such articles of food and in the viscera of those persons who have succumbed. In other cases, such as in high game, cooking may kill the micro-organisms, but does not necessarily destroy the chemical products of putrefaction. The well-known poisonous properties of mussels appear to be due to the production of a ptomaine when these shell-fish have been lying in water polluted by sewage. If such poisonous mussels are taken out to the open sea and laid down for a few months they lose their poisonous properties. A poisonous substance known as tyrotoxicon may also develop in milk, and the same substance may be present in cheese, and lead to severe symptoms and even death.

[1] Lancet, March 31, 1894, p. 836. [2] Med. Record, Aug. 4, 1894, p. 133. [3] Med. Record, Oct. 6, 1894, p. 436. [4] Med. Record, Aug. 18, 1894. [5] Med. Record, p. 113, July 28, 1894; Bult. Gen. de Therapeut., May 30, 1894. [6] Clin. Journal, April, 1894, p. 367. [7] B M. J., June 23, 1894. [8] Lancet, Oct 20, 1884. [9] Med. Week, June 1, 1894. [10] Med. Record, June 16, 1894. [11] New York Med. Journal, Nov. 3, 1894. [12] B. M. J., Sept. 29, 1894. [13] Pharmaceut. Journal, Mar. 3, 1894.

